# From freehand to precision: Dynamic navigation systems in transmandibular nerve canal implantation: A case series

**DOI:** 10.1097/MD.0000000000041922

**Published:** 2025-03-14

**Authors:** Shiwei Che, Noor Huda Ismail, Wuxiang Wang, Raja Azman Awang

**Affiliations:** a School of Dental Sciences, Health Campus, Universiti Sains Malaysia, Kelantan, Malaysia; b Dr. Che Dental Clinic, Luzhou City, China; c Prosthodontics Unit, School of Dental Sciences, Health Campus, Universiti Sains Malaysia, Kelantan, Malaysia; d Department of Trauma Surgery, The First People’s Hospital of Zhaotong, Zhaotong City, Yunnan Province, China; e Periodontics Unit, School of Dental Sciences, Health Campus, Universiti Sains Malaysia, Kelantan, Malaysia.

**Keywords:** dental implantation, dynamic navigation systems, inferior alveolar nerve, surgical guides, transmandibular nerve canal

## Abstract

**Rationale:**

This case series explores dynamic navigation systems (DNS) in transmandibular nerve canal implantation for patients with limited jaw space due to the proximity of the inferior alveolar nerve (IAN). The aim is to demonstrate how DNS improves implant placement precision and safety in cases where traditional methods face challenges.

**Patient concerns:**

The patients had limited bone height and proximity to the IAN, making traditional implant techniques challenging. Concerns included the risk of nerve damage and difficulties in achieving optimal implant placement due to anatomical constraints.

**Diagnoses:**

Both patients had severe bone resorption and insufficient bone height in the posterior mandible, with concerns about IAN positioning. The first patient had grade III mobility in tooth #46 with a periapical infection, while the second had bilateral posterior mandibular pain and grade III mobility in tooth #47.

**Interventions:**

Implants were placed using DNS, allowing precise planning and real-time guidance during surgery. Based on cone beam computed tomography, preoperative planning assessed bone height and IAN proximity. DNS ensured accurate implant placement, avoiding nerve interference, while bone grafts and growth factors were applied for healing.

**Outcomes:**

Both cases showed successful implant placement without complications like nerve damage or implant misplacement. Follow-up cone beam computed tomography scans confirmed well-positioned implants, with minimal bone resorption in the first case over 2 years and stable conditions in the second case after 6 months.

**Lessons:**

This series highlights DNS’s effectiveness in improving implant accuracy and reducing nerve injury risks, suggesting its value in complex dental implant surgeries.

## 1. Introduction

Dental implantation has evolved into the primary therapy for replacing posterior mandibular molars after their loss, as it significantly improves the ability to chew. Nevertheless, the distinctive structural limitations of the back part of the lower jaw, such as the close proximity of the inferior alveolar nerve (IAN), pose significant difficulties for the traditional implant methods. To guarantee the effectiveness and security of dental implants in these regions, several tactics are utilized, depending on the amount of bone height above the IAN. Standard-length implants (≥10 mm) can be used immediately if there is sufficient bone height (≥10 mm). For cases of moderate bone shortages measuring between 7 and 10 mm, possible solutions include the use of short implants that vary in length from 6 to 8 mm or the implementation of angled implantation procedures.^[1,2]^ When there is a moderate shortage of bone (4–6 mm), it may be necessary to perform bone grafting, alveolar ridge augmentation, or ultrashort implants (4–6 mm). If there are substantial inadequacies (<4 mm), more intricate treatments such as nerve relocation or extensive bone augmentation techniques such as only grafting or distraction osteogenesis may be necessary.^[2]^

Research has indicated that a considerable proportion of websites have bone heights below 7 mm, which poses a challenge for the utilization of normal implant lengths. Indeed, up to 40% of these locations may not accommodate typical implants because of inadequate vertical bone height.^[3,4]^ While traditional methods can successfully enhance bone volume, they typically require intricate surgical procedures and lengthy recuperation periods, which place significant physical and psychological strain on patients. Thorough preoperative assessments using cone beam computed tomography (CBCT) are essential for evaluating bone quality and soft tissues as well as for assessing risks and consulting with patients, particularly in cases involving nerve relocation or complex procedures.^[5]^

When dealing with cases in which there is no more than 7 mm of bone over the mandibular nerve canal, 1 possible treatment option is crossing the canal for implant insertion.^[2]^ This method is especially appropriate when conventional implant methods are not possible, enabling secure and efficient placement of implants in cases of restricted bone height, while reducing the risk of harm to the IAN. Methods such as inclined placement of implants and utilization of abbreviated implants are successful in tackling these difficulties.^[2,6]^ In addition, the application of dynamic navigation systems (DNS) improves accuracy and safety by allowing real-time monitoring throughout the implantation procedure, ensuring precise positioning, and preventing damage to the mandibular nerve.^[7]^

## 2. Case reports

### 2.1. Case 1

**Figure 1.**
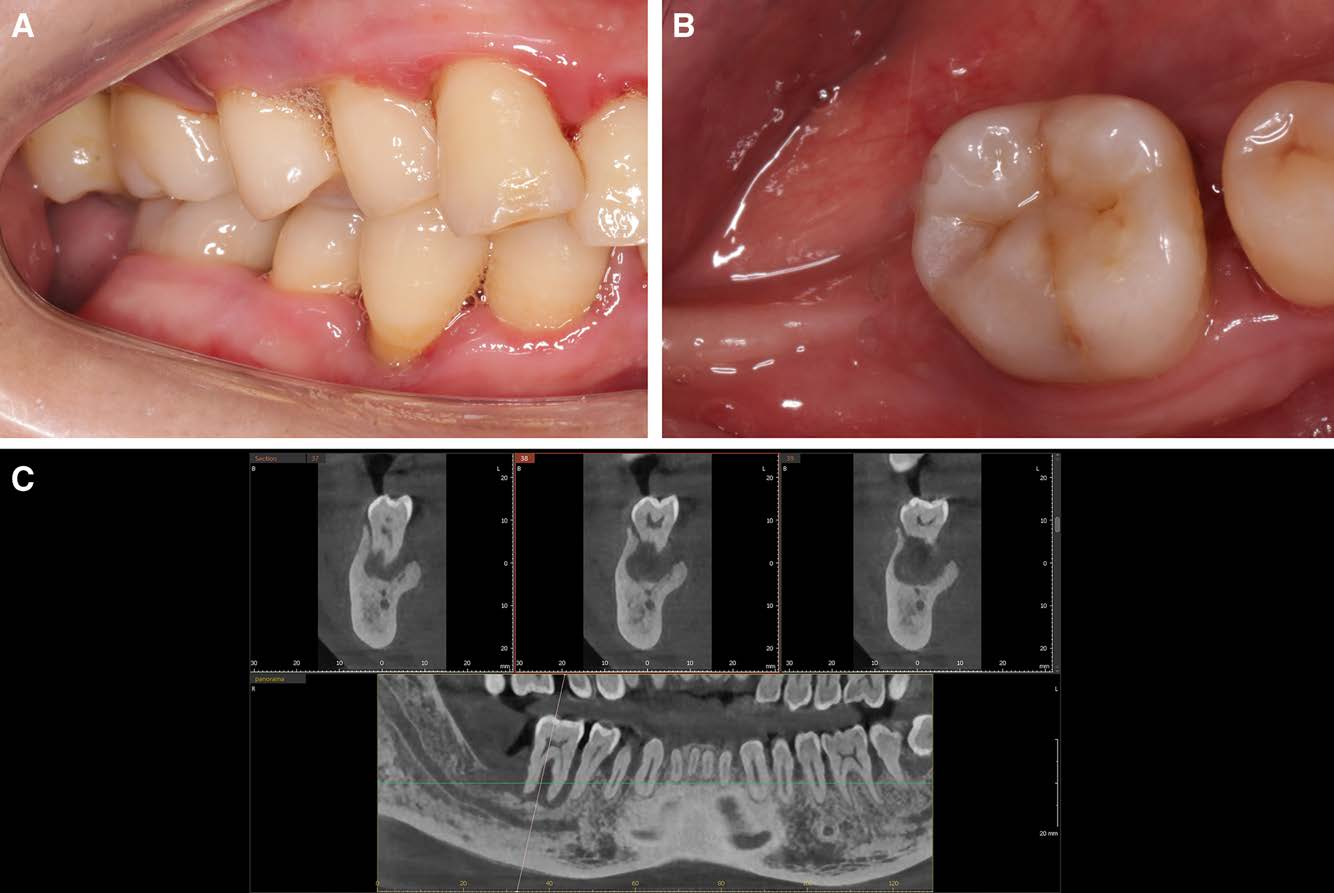
(A) View of the buccal side of the right posterior area; (B) view of the occlusal surface of tooth #46 right lower molar area; (C) preoperative assessment for tooth #46 using cone beam computed tomography (CBCT) scan.

A 36-year-old Chinese male presented with grade III mobility in tooth #46 and missing tooth #47, accompanied by generalized mild gingivitis and significant gingival recession in multiple areas (Fig. 1A and B). Preoperative CBCT scans demonstrated pronounced low-density radiographic features around the apical region of tooth #46, with severe alveolar bone resorption in regions #46 and #47 (Fig. 1C). The patient smoked 10 cigarettes per day and had no systemic diseases.

Considering the absence of teeth in the lower jaw, dental implantation is the most favored approach to effectively restore the ability to function and chew. Extraction of tooth #46 was necessary because of the acute periapical infection, which could not be adequately treated with integrated periodontal and endodontic procedures, and the significant loss of alveolar bone in this area.

This operation was performed 1 week after thorough periodontal cleaning, principally aimed at temporarily stabilizing the periodontal state before extraction. Merely performing comprehensive periodontal cleaning is not sufficient to effectively cure active periodontal disease observed on CBCT. A more extensive periodontal treatment plan is required to guarantee long-term stability of periodontal health. To prevent frequent issues that occur after tooth extraction, such as loss of alveolar bone, site preservation measures were promptly used after tooth removal. These treatments, which entail inserting graft material into the extraction socket to uphold the alveolar ridge and prevent its collapse, are vital for preserving bone structure and are necessary for future dental implant installation.

**Figure 2.**
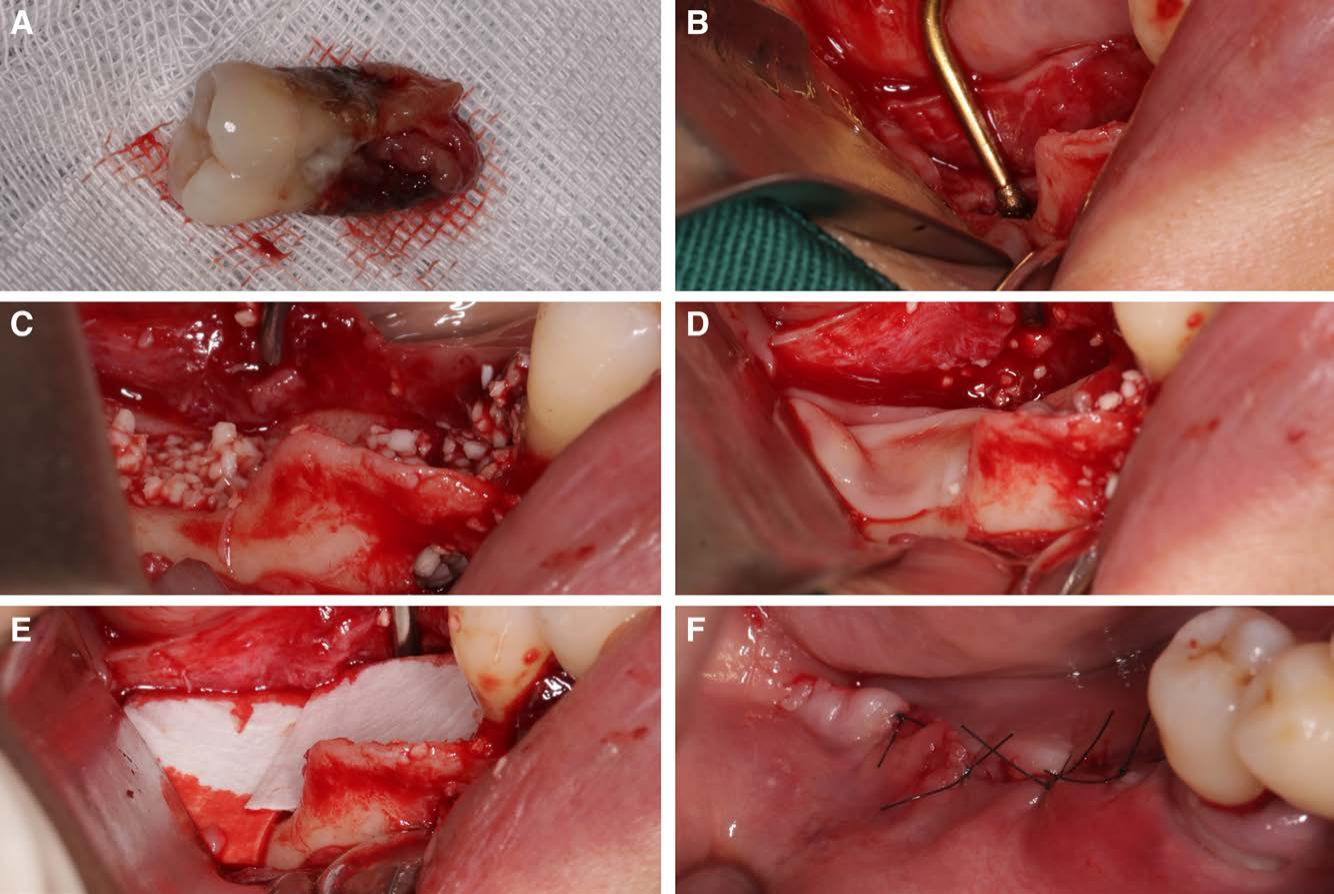
(A) Extracted tooth #46; (B) cleansing of the extraction socket using piezosurgery; (C) placement of Bio-Oss® (Geistlich, Switzerland) bone graft material; (D) concentrated growth factors (CGF) is placed in the socket; (E) oral absorbable biomembrane (Megreen, China) is placed on the surface; (F) closure of the socket with 4-0 nylon sutures.

Under local anesthesia with 4% articaine hydrochloride, tooth #46 was extracted (Fig. 2A). The granulation tissue present in the extraction socket was carefully eliminated using a curette and then irrigated with saline solution. The socket was subsequently cleaned using an ultrasonic bone scalpel in conjunction with chilling saline solution to reveal the newly exposed bone surfaces (Fig. 2B). The socket was filled with Bio-Oss® (Geistlich, Switzerland) bone graft material (Fig. 2C). Concentrated growth factors (CGF), obtained from autologous blood via differential centrifugation performed preoperatively, CGF, obtained from autologous blood via differential centrifugation performed preoperatively, were then added to enhance regeneration (Fig. 2D). An oral absorbable biomembrane (Megreen, China) was used to cover the membrane (Fig. 2E). Finally, the site was sutured with 4-0 nylon sutures (Fig. 2F) to promote optimal healing and to preserve the site post-extraction.

**Figure 3.**
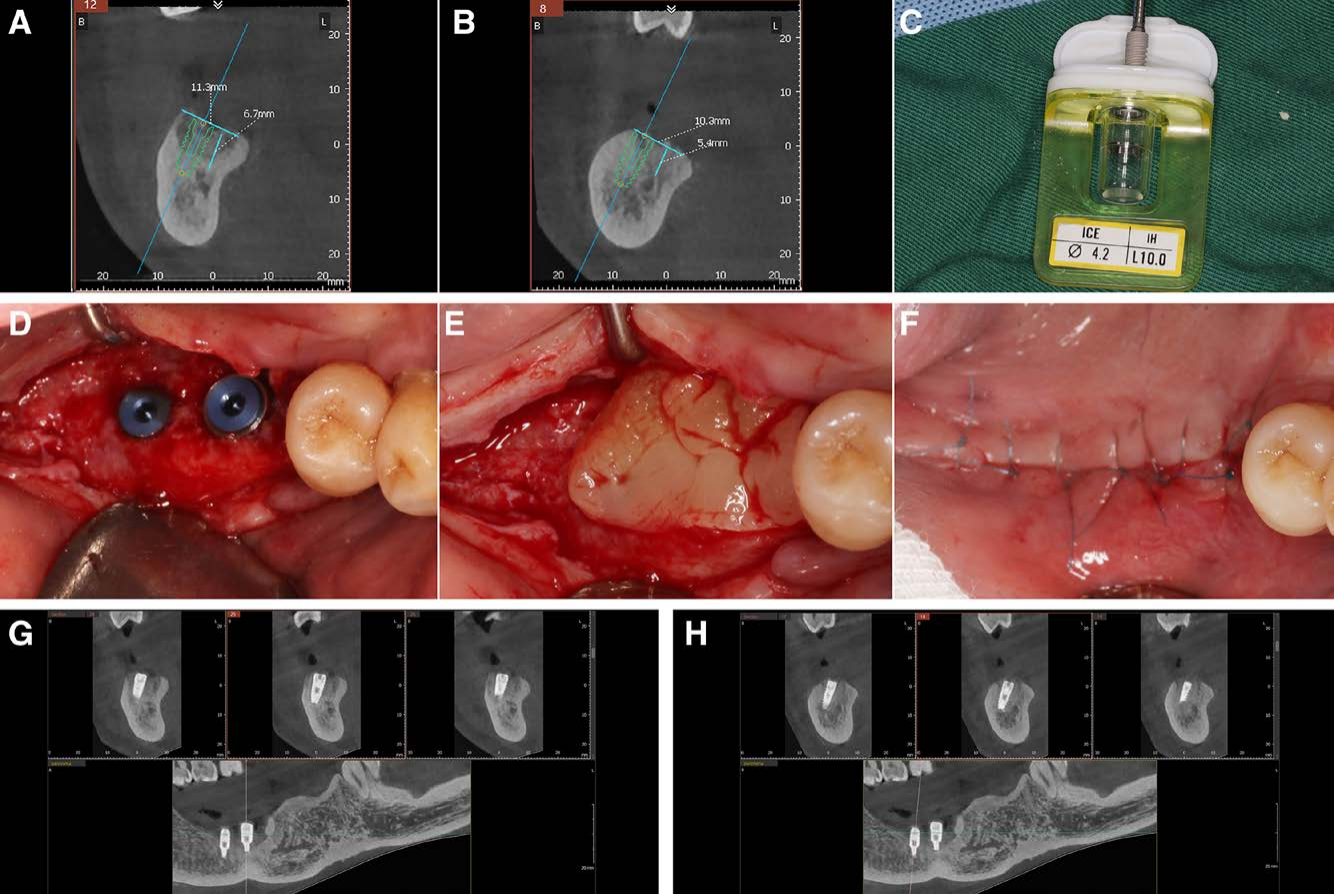
(A) CBCT imaging and simulated implant placement at site #46, 4 months post-extraction; (B) CBCT imaging and simulated implant placement at site #47; (C) Alpha-Bio Tec, ICE, ABT1020 implant (diameter 4.2 mm, length 10 mm); (D) implant placement at sites #46 and #47; (E) application of concentrated growth factors (CGF); (F) closure of the site with 5-0 nylon sutures; (G) postimplantation CBCT scan of tooth #46; (H) postimplantation CBCT scan of tooth #47. CBCT = cone beam computed tomography.

After 4 months of healing, the residual bone height above the IAN canal at site #46 was measured using CBCT and found to be approximately 6.7 mm, with a width of approximately 11.3 mm (Fig. 3A). In addition, at site #47, the height was about 5.4 mm with a width of 10.3 mm (Fig. 3B). According to the ITI implant guidelines, a vertical bone height of <7 mm may necessitate bone grafting, alveolar ridge augmentation, nerve repositioning, or the use of ultrashort implants.^[8]^ However, considering the adequate bone width and sufficient keratinized gingiva on the buccal side in this region, a simulation was performed using CBCT software with 2 implants, each having a diameter of 4.2 mm and length of 10 mm (Fig. 3A and B). This simulation revealed that oblique implant placement (with a maximum angle of 30°) across the nerve canal could successfully achieve a normal implant restoration. This approach simplifies the procedural steps, reduces the psychological stress on the patient, and shortens the surgical period.

Before surgery, the patient was administered 500 mg of oral amoxicillin 3 times a day, starting 1 day before the procedure, to prevent infection. The surgical area was routinely disinfected and draped. Local anesthesia was achieved by injecting 4% articaine hydrochloride into the surgical site. A full-thickness mucoperiosteal flap is elevated during the procedure. Implant site preparation was carefully performed using the Alpha-Bio implant toolkit according to the manufacturer’s guidelines. The pilot holes were cautiously angled as preoperatively planned and gradually enlarged with ample cooling provided by the chilled saline solution. Bone integrity was checked frequently using a probe to ensure that the IAN canal was not breached.

After preparing the sites, implants (Alpha-Bio Tec, ICE, ABT1020A) were placed at positions 46 and 47, achieving a final insertion torque≥35 N cm (Fig. 3C and D). Cover screws were installed, followed by application of CGF obtained from autologous blood via differential centrifugation (Fig. 3E). The sites were meticulously closed using 5-0 nylon sutures to ensure proper healing (Fig. 3F). Immediate postoperative CBCT confirmed that the implants were well positioned and closely aligned with the preoperative plan (Fig. 3G and H). Twelve days after surgery, during suture removal, no complications, such as numbness of the lower lip or other signs of IAN damage, were observed.

**Figure 4.**
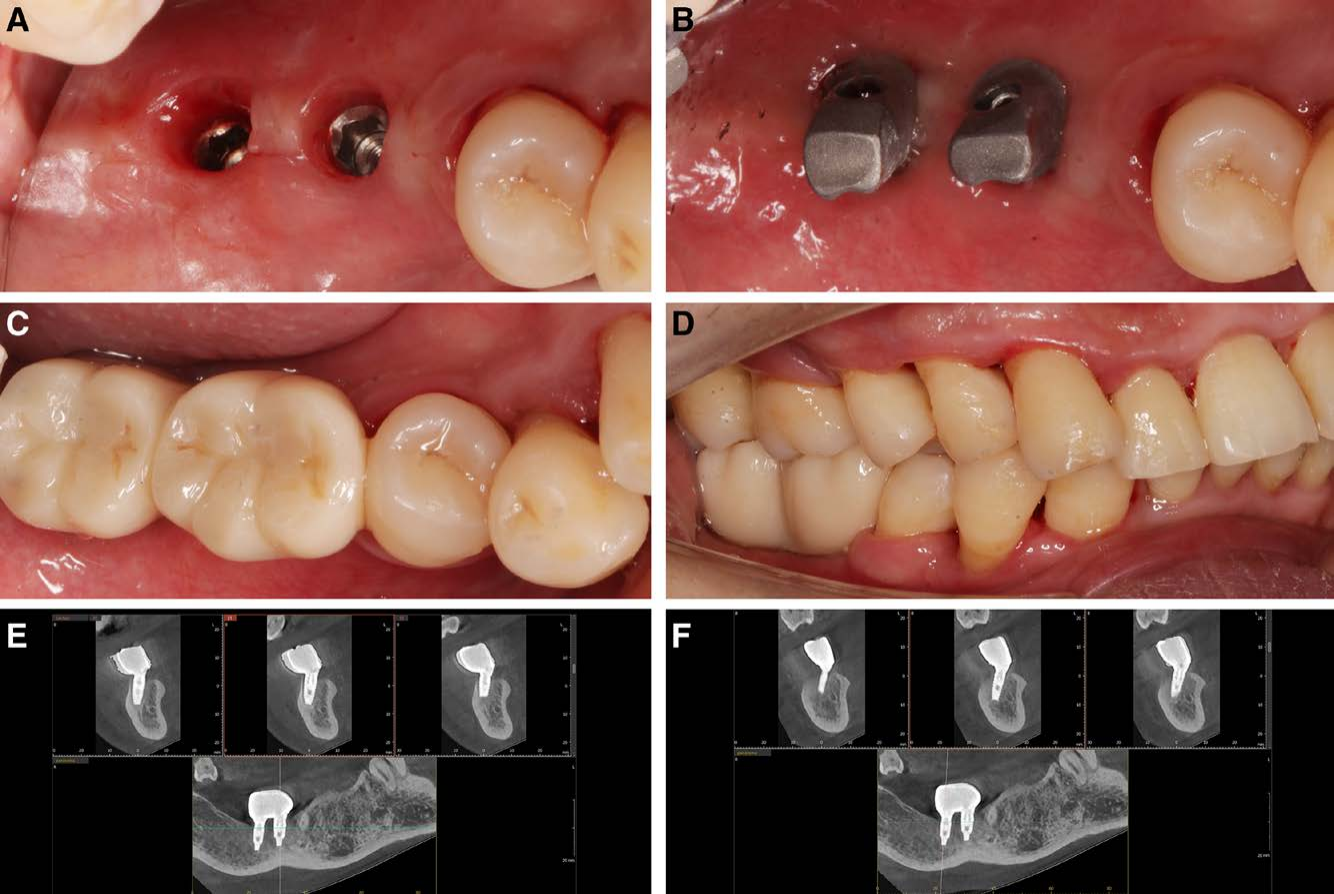
(A) Intraoral photo 4 months post-operation, after soft tissue contouring using a healing abutment; (B) personalized final restoration abutment fitted 4 months post-operation; (C) final restoration photo; (D) occlusal view of the final prosthetic crowns in place; (E) follow-up CBCT image of site #46, 2 years after implant restoration; (F) follow-up CBCT image of site #47, 2 years after implant restoration. CBCT = cone beam computed tomography.

Three months postoperatively, second-stage implant surgery was performed at sites #46 and #47, where healing abutments were placed (Fig. 4A). Four months after surgery, soft tissue contouring was completed and implant-supported crowns were fabricated, finalizing the restoration (Fig. 4B–D). Two years after completion of the final restoration, follow-up CBCT imaging showed minimal alveolar bone resorption with no other abnormalities (Fig. 4E and F). Throughout the 2-year postoperative period, normal function was maintained in patients #46 and #47, achieving a favorable prognosis.

### 2.2. Case 2

**Figure 5.**
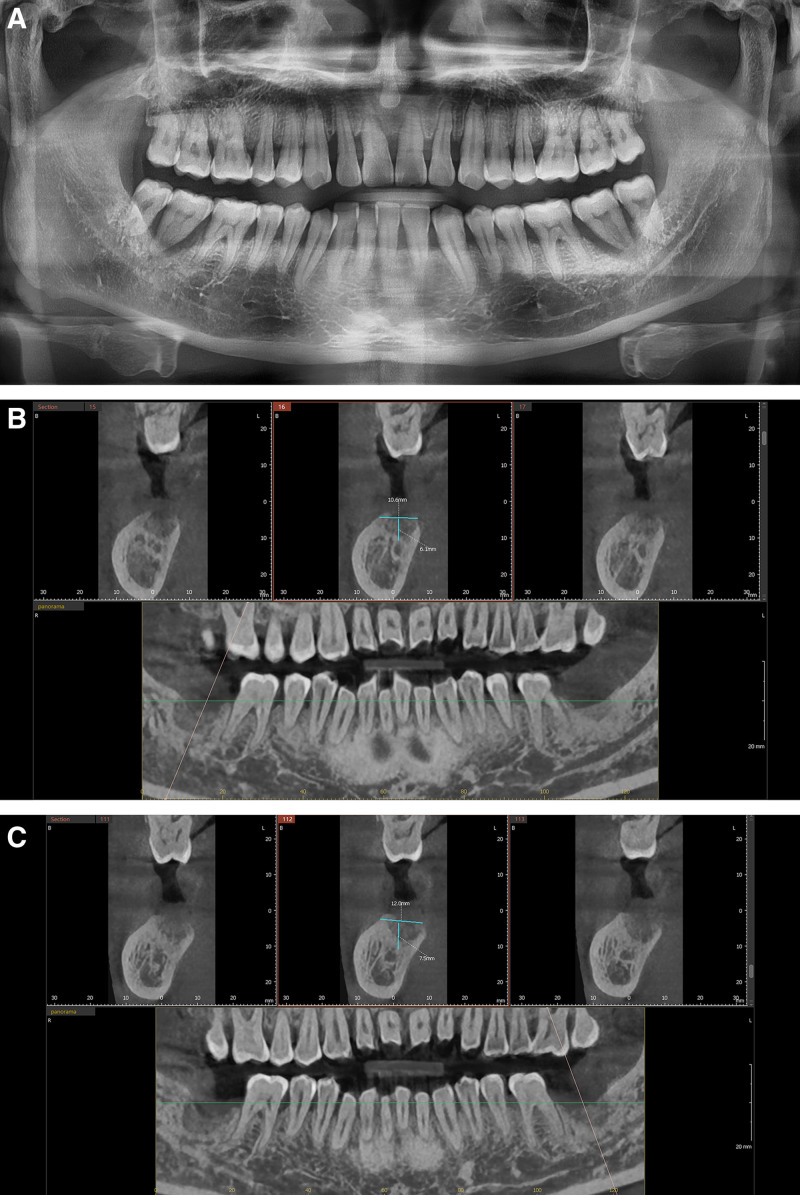
(A) Initial panoramic radiograph; (B) CBCT of site #47, 4 months post-extraction; (C) CBCT of site #37, 4 months post-extraction. CBCT = cone beam computed tomography.

A 42-year-old Chinese male had experienced bilateral posterior mandibular pain several months after chewing. Clinical examination showed grade III mobility of tooth #47, grade I mobility of tooth #48, and grade III mobility of tooth #37. Initial panoramic imaging revealed periapical radiolucency encompassing the entire root of teeth #47 and #38 (Fig. 5A). The patient, who smoked approximately 10 cigarettes daily, had no history of systemic diseases.

Given the severe periapical inflammation and inability to restore function through endodontic and periodontal treatments, teeth #47, #48, and #37 were extracted under local anesthesia, with future consideration for implant restoration for #47 and #37. Four months post-extraction, CBCT imaging showed residual alveolar bone heights of approximately 6.1 mm at site #47 (Fig. 5B) and 7.5 mm at site #37, with crest widths of 10.6 and 12 mm, respectively (Fig. 5C). Despite healing for over 4 months, CBCT still showed low-density radiolucency and sparse trabeculation in the extraction sockets at both sites.

Similar to patient #1, simulated implant placement at site #47 using CBCT software suggests the feasibility of crossing the nerve canal. Considering the remaining alveolar height of ≥7 mm at site #37, which is suitable for ultrashort implants, an ultrashort implant was planned for this site.^[9]^ To ensure precision and safety throughout the procedure, the entire surgical plan involved implant placement guided by DNS.

**Figure 6.**
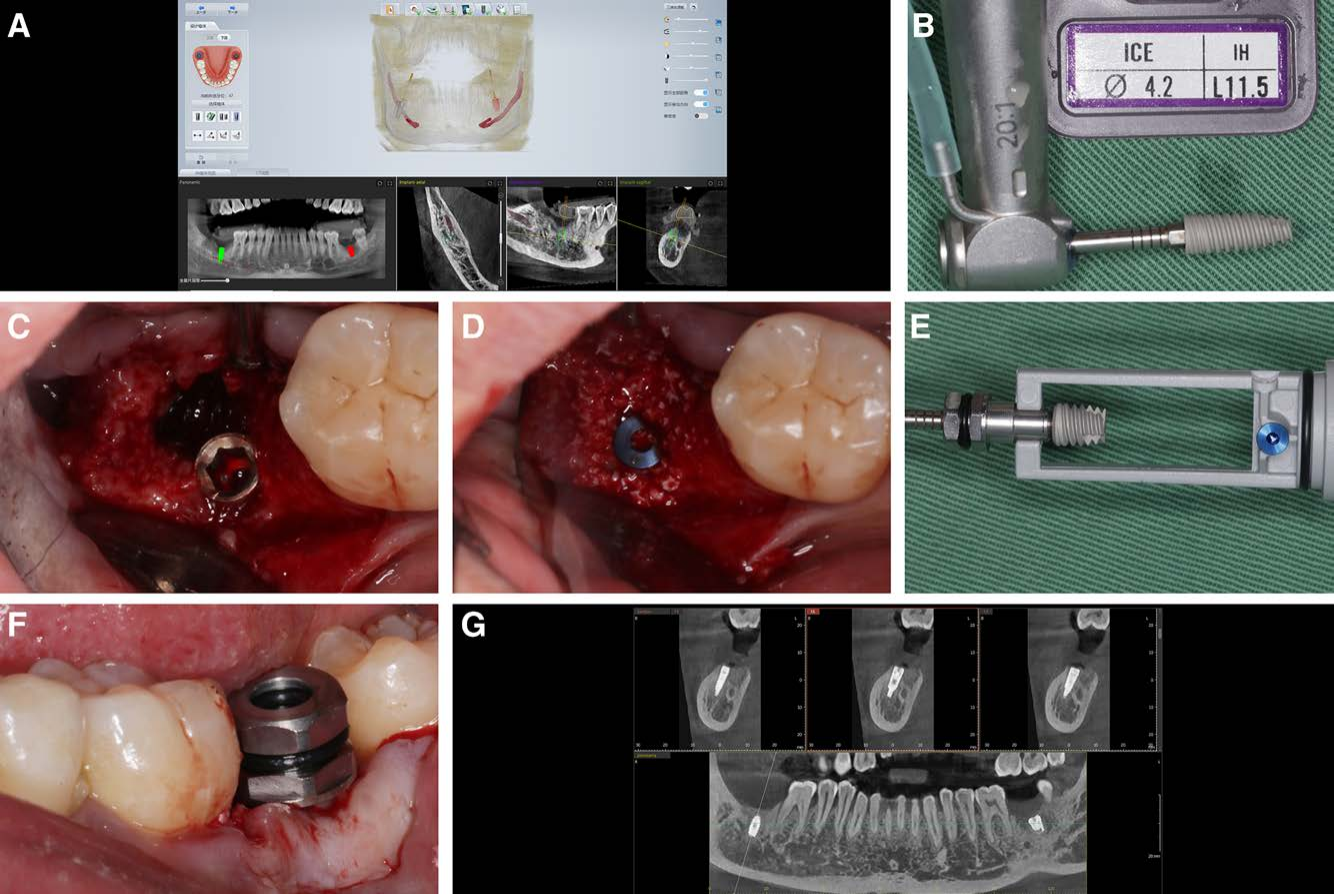
(A) Preoperative planning using the dynamic navigation system (DCARER, China); (B) Alpha-Bio Tec, ICE, ABT1021 implant (diameter 4.2 mm, length 11.5 mm); (C) implant placement at site #46; (D) filling the gap between the implant and bone wall with Bio-Oss® (Geistlich, Switzerland) bone graft material; (E) Alpha-Bio Tec, ICE, ABT1021 implant (diameter 4.2 mm, length 11.5 mm); (F) Alpha-Bio Tec, ATID implant (diameter 5 mm, length 6 mm); (G) immediate postoperative CBCT.

A registration device and a U-shaped tube were installed in the edentulous area, and CBCT was performed using a VATECH PHT-35LH5 (Korea). The U-shaped tube was then removed and preserved. The CBCT data were imported into a DNS (DCARER, China) for three-dimensional reconstruction (Fig. 6A).

The distance from the IAN canal to the crest of the alveolar ridge and the buccal bone thickness adjacent to the nerve canal were measured. The virtual implant placement was adjusted such that the implant was positioned between the IAN and buccal outer plate. The implant was set at a minimum distance of 1.5 mm from the IAN and a minimum of 1.5 mm from the bone on the lateral side of the implant to ensure safety and structural integrity. Implant placement was optimized to the ideal position and the preoperative plan was saved for future reference.

Before the procedure, the patient was administered oral amoxicillin and the surgical area was routinely disinfected and draped. navigation device was positioned approximately 1.25 to 1.5 mm in front of the patient’s head on the left side, the instruments were connected, and the reference board was calibrated. A self-curing resin was used to install and secure the fixture, and the U-shaped registration device was reinstalled in the edentulous area to commence registration, aiming for an error within 0.3 mm; after successful registration, the U-shaped tube was removed.

Local infiltration anesthesia with 4% articaine hydrochloride was administered in the implant area, and a small mesiodistal incision slightly larger than the diameter of the implant was made at the crest of the alveolar ridge (a regular incision was required for simultaneous bone grafting to fully expose the implant site). The mucoperiosteum was buccolingually reflected to expose the bone surface of the alveolar crest at the implant site. Under the guidance of real-time DNS, following the preoperative plan, implant surgery was performed using the Alpha-Bio implant toolkit, according to its guidelines. Gradual drilling was completed, and an implant (Alpha-Bio Tec, ICE, ABT1021; diameter 4.2 mm, length 11.5 mm) was placed at site #47 (Fig. 6A–C). The gap between the implant and bone wall was filled with Bio-Oss® (Geistlich, Switzerland) bone graft material (Fig. 6D). At site #37, an Alpha-Bio Tec ATID implant (diameter, 5 mm; length, 6 mm) was inserted, and the procedure was concluded with suturing (Fig. 6E and F). Immediate postoperative CBCT showed that implants at sites 46 and 37 were positioned almost exactly as planned, confirming that the implants were ideally placed (Fig. 6G).

**Figure 7.**
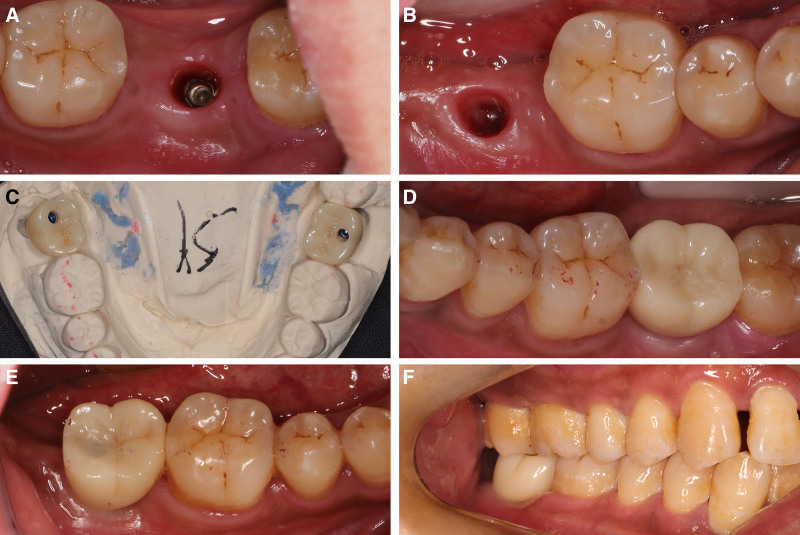
(A) Post-gingival contouring with a healing abutment in place for tooth #37; (B) post-gingival contouring with a healing abutment in place for tooth #47; (C) dental model of the upper prosthetic restoration for teeth #47 and #37; (D) intraoral view of the final restoration for tooth #37; (E) intraoral view of the final restoration for tooth #47; (F) occlusal view of the final restoration for tooth #47.

During the 4-month follow-up recovery period, there were no symptoms of numbness in the lower lip or damage to the IAN in implant-related areas. In the 5th month postoperatively, after completing gingival contouring with healing abutments, prosthetic crown restorations were performed (Fig. 7A–C). The final restorations at sites #37 and #47 were completed after crown placement (Fig. 7D–F).

**Figure 8.**
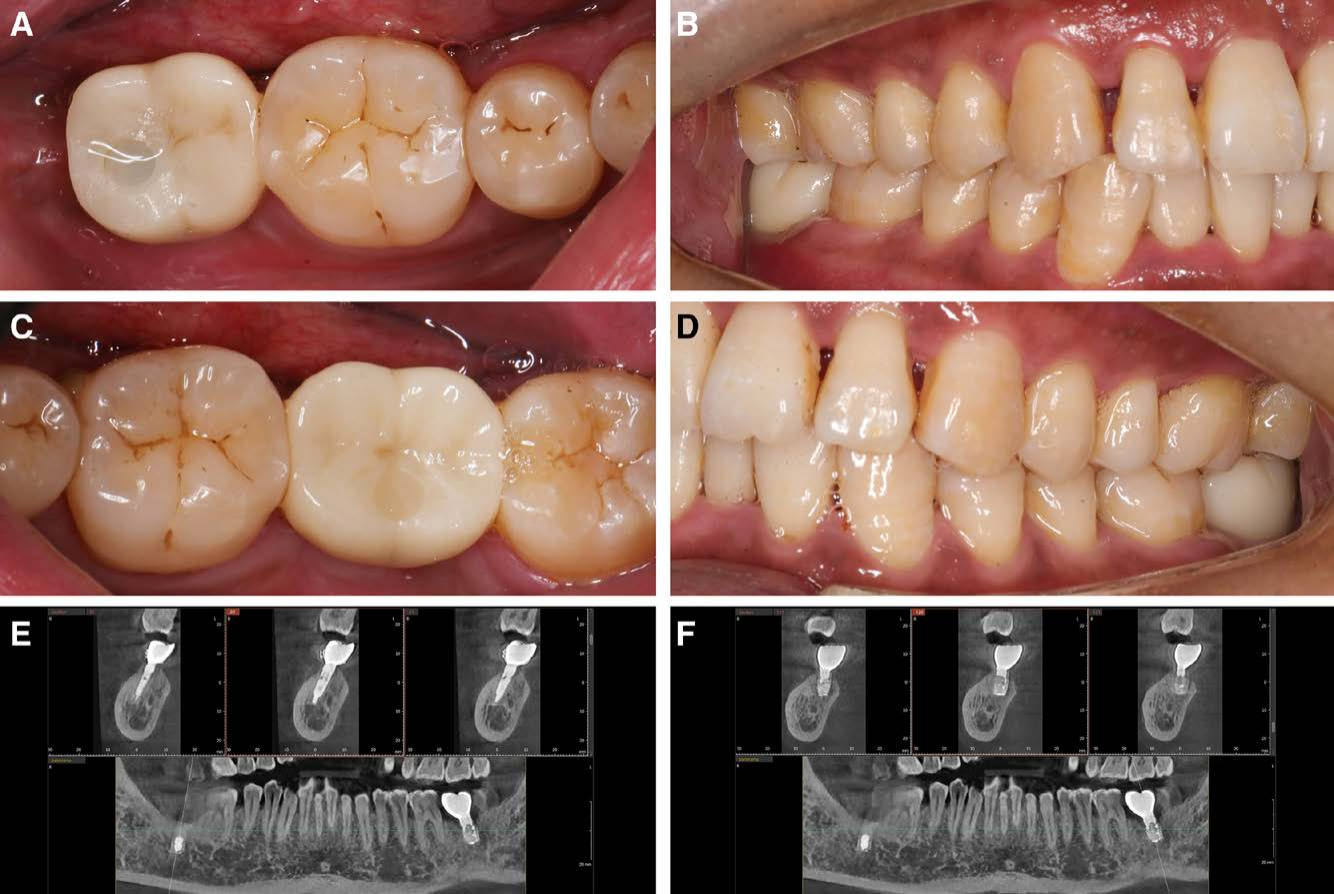
(A) Intraoral view at the 6-month follow-up after the final restoration of #47; (B) occlusal view at the 6-month follow-up after the final restoration of #47; (C) intraoral view at the 6-month follow-up after the final restoration of #37; (D) occlusal view at the 6-month follow-up after the final restoration of #37; (E) CBCT image at the 6-month follow-up after the final restoration of #47; (F) CBCT image at the 6-month follow-up after the final restoration of #37. CBCT = cone beam computed tomography.

During the 6-month follow-up after the final restoration, clinical examination showed that teeth #37 and #47 were in good condition intraorally and functioned normally (Fig. 8A–D). Follow-up CBCT scans also indicated stable status for implants #37 and #47 (Fig. 8E and F).

## 3. Discussion

Transmandibular nerve canal implantation is recommended for individuals with extensive posterior mandibular atrophy who cannot receive standard implants because of major anatomical restrictions. Anatomical studies have demonstrated that the mandibular canal frequently changes its path from being closer to the cheek to being closer to the tongue, and from the first premolar to the third molar. This movement allows more bone to be available on the cheek side at the location of the second molar. This anatomical characteristic enables the placement of transmandibular implants on the buccal side of the second molar area. This region typically has a buccal bone width of approximately 6.913 ± 1.222 mm, making it a favorable location for this procedure.^[10]^

Transmandibular implantation may circumvent the necessity of substantial bone grafting or nerve repositioning; however, it is associated with the inherent risk of nerve damage. If not performed cautiously, it can result in postoperative numbness.^[11]^ Standard practice suggests maintaining a minimum safe distance of 2 mm from the mandibular nerve to prevent such difficulties. However, recent research has shown that a distance of <2 mm may not always lead to nerve damage.^[12]^ Biomechanical analyses have recommended a minimum safe distance of approximately 1.0 mm. However, in clinical settings, a margin of 1.5 mm is used to accommodate for surgical mistakes. Moreover, the extent of keratinized gingiva plays a vital role in ensuring enduring stability and well-being of implants. It is generally advised to have a minimum width of 2 mm to create an effective seal of the soft tissue, which helps minimize the chances of infection and irritation.

Ultrashort implants, with a length as small as 6 mm, can be used in regions with limited vertical bone height to optimize the utilization of the existing bone. These implants have demonstrated satisfactory survival rates, albeit with a slightly elevated chance of failure compared to standard-length implants.^[13]^ The guidelines for prosthetic restoration of these implants emphasize the significance of preventing excessive strain on both the bone and the implant. Biomechanical research indicates that the most favorable inclination angle should be <30° in order to reduce biomechanical drawbacks.^[14]^

DNS provides a notable improvement in the accurate positioning of implants, particularly in intricate scenarios such as crossing the transmandibular nerve canal. This technology enables immediate surgical guidance, minimizes the possibility of deviations from the intended implant trajectory, and improves overall treatment results. Although traditional surgical guides offer a certain degree of accuracy, they may not provide the same level of real-time flexibility and adaptability as DNS.^[15]^

Transmandibular treatment often involves freehand implant insertion, which is a widespread practice. However, this technique significantly depends on the clinician’s expertise and is therefore very sensitive to the technical skills required for its execution. In the absence of accurate preoperative imaging and meticulous planning, there is a substantial likelihood of nerve injury resulting from implant placement errors. DNS and surgical guides greatly mitigate these dangers while enhancing precision, a crucial factor considering their close proximity to vital anatomical systems, such as the mandibular nerve.

In summary, considering the achievements of the aforementioned instances and prior studies, transmandibular nerve canal implantation is a viable alternative for individuals with notable anatomical constraints. Nevertheless, careful and detailed preoperative preparation and accurate implementation are required to ensure safety and efficacy. DNS and surgical guides are indispensable instruments that significantly improve the precision of implant placement, potentially decreasing the occurrence of problems associated with freehand approaches.

## 4. Conclusions

The transmandibular nerve canal implant technique is feasible in patients with anatomical constraints. Additionally, the use of DNS enhances the precision and safety of dental implant procedures, especially in patients with limited bone height.

## Author contributions

**Conceptualization:** Shiwei Che.

**Formal analysis:** Noor Huda Ismail.

**Investigation:** Wuxiang Wang.

**Methodology:** Shiwei Che.

**Project administration:** Noor Huda Ismail.

**Resources:** Shiwei Che.

**Supervision:** Raja Azman Awang.

**Validation:** Raja Azman Awang.

**Visualization:** Wuxiang Wang.

**Writing – original draft:** Shiwei Che.

**Writing – review & editing:** Raja Azman Awang.

## References

[1] Jung RE Al-Nawas B Araujo M. Group 1 ITI consensus report: the influence of implant length and design and medications on clinical and patient-reported outcomes. Clin Oral Implants Res. 2018;29(Suppl 16):69–77. 30328189 10.1111/clr.13342

[2] Sghaireen MG Srivastava KC Shrivastava D. A CBCT based three-dimensional assessment of mandibular posterior region for evaluating the possibility of bypassing the inferior alveolar nerve while placing dental implants. Diagnostics (Basel). 2020;10:406. 32545908 10.3390/diagnostics10060406 PMC7344927

[3] Braut V Bornstein M Kuchler U Buser D. Bone dimensions in the posterior mandible: a retrospective radiographic study using cone beam computed tomography. Part 2—analysis of edentulous sites. Int J Periodontics Restorative Dent. 2014;34:639–47. 25171034 10.11607/prd.1895

[4] Bressan E Ferrarese N Pramstraller M Lops D Farina R Tomasi C. Ridge dimensions of the edentulous mandible in posterior sextants: an observational study on cone beam computed tomography radiographs. Implant Dent. 2017;26:66–72. 27824716 10.1097/ID.0000000000000489

[5] Deryabin G Grybauskas S. Dental implant placement with inferior alveolar nerve repositioning in severely resorbed mandibles: a retrospective multicenter study of implant success and survival rates, and lower lip sensory disturbances. Int J Implant Dent. 2021;7:44. 34105021 10.1186/s40729-021-00334-x PMC8187674

[6] Sivolella S Meggiorin S Ferrarese N. CT-based dentulous mandibular alveolar ridge measurements as predictors of crown-to-implant ratio for short and extra short dental implants. Sci Rep. 2020;10:16229. 33004827 10.1038/s41598-020-73180-3 PMC7530749

[7] Yu X Tao B Wang F Wu Y. Accuracy assessment of dynamic navigation during implant placement: a systematic review and meta-analysis of clinical studies in the last 10 years. J Dent. 2023;135:104567. 37263412 10.1016/j.jdent.2023.104567

[8] Wismeijer D Chen ST. Proceedings of the 6th ITI consensus conference. Clin Oral Implants Res. 2018;29:5–7. 30328198 10.1111/clr.13301

[9] Lombardo G Signoriello A Pardo A. Short and ultra-short (<6-mm) locking-taper implants supporting single crowns in posterior areas (part II): a 5-year retrospective study on periodontally healthy patients and patients with a history of periodontitis. Clin Implant Dent Relat Res. 2022;24:455–67. 35635514 10.1111/cid.13103 PMC9546440

[10] Jinming L Wenli W Lingyi D Ming S. Radiological measurement and analysis of trans-inferior alveolar nerve implantation. Stomatology. 2023;2:130–4.

[11] Szalma J Vajta L Lempel E Tóth A Jeges S Olasz L. Intracanal temperature changes during bone preparations close to and penetrating the inferior alveolar canal: drills versus piezosurgery. J Craniomaxillofac Surg. 2017;45:1622–31. 28843405 10.1016/j.jcms.2017.07.007

[12] Lin MH Mau LP Cochran DL Shieh YS Huang PH Huang RY. Risk assessment of inferior alveolar nerve injury for immediate implant placement in the posterior mandible: a virtual implant placement study. J Dent. 2014;42:263–70. 24394585 10.1016/j.jdent.2013.12.014

[13] Rossi F Lang NP Ricci E Ferraioli L Baldi N Botticelli D. Long-term follow-up of single crowns supported by short, moderately rough implants—a prospective 10-year cohort study. Clin Oral Implants Res. 2018;29:1212–9. 30430655 10.1111/clr.13386

[14] Anitua E de Ibarra NLS Martín IM Rotaeche LS. Influence of implant tilting and length on the biomechanics of single-tooth restoration: a finite element analysis in atrophic mandible. Dent J. 2022;10:77. 10.3390/dj10050077 PMC9139222 35621530

[15] Geng N Ren J Zhang C Zhou T Feng C Chen S. Immediate implant placement in the posterior mandibular region was assisted by dynamic real-time navigation: a retrospective study. BMC Oral Health. 2024;24:208. 38336661 10.1186/s12903-024-03947-x PMC10858590

